# Executive functions and personality traits of juvenile myoclonic epilepsy patients: a single-center experience of 23 cases

**DOI:** 10.55730/1300-0144.5354

**Published:** 2021-12-21

**Authors:** Berin GÜLATAR TÜRKOĞLU, İpek MİDİ, Kadriye AĞAN YILDIRIM

**Affiliations:** Department of Neurology, Faculty of Medicine, Marmara University, İstanbul, Turkey

**Keywords:** Juvenile myoclonic epilepsy, executive functions, temperament and character inventory

## Abstract

**Background/aim:**

Juvenile myoclonic epilepsy (JME), which is a fairly common form of generalized epilepsy syndrome has attracted attention by providing focal findings in some electrophysiological, neuropsychological, and neuroimaging studies. These findings are considered to be based on frontal lobe dysfunction. Furthermore, it is known that Cluster B personality disorders that are related to impulsive behavior are frequently seen in JME patients.

**Materials and methods:**

In this study, 23 JME patients and 20 healthy control subjects were included. All subjects were assessed using neuropsychological tests for executive functions and the Temperament and Character Inventory (TCI) for personality traits.

**Results:**

JME patients performed poorly in the digit span test and the Stroop Color and Word Interference Test. When the TCI scores were compared, there was no significant difference between the patients and the control subjects compatible with the literature. In addition, cooperativeness-character dimension (C1-social acceptance) scores were significantly lower in the patient group.

**Conclusion:**

Our findings support that JME patients have frontal lobe dysfunction. Although several studies are available in the literature, no significant results related to personality traits were detected.

## 1. Introduction

Juvenile myoclonic epilepsy (JME) is characterized by a triad of myoclonic jerks, in all patients, generalized tonic-clonic (GTC) seizures in more than 90%, and typical absence seizures in nearly 30% [1]. Intelligence levels of patients and routine cranial magnetic resonance imaging (MRI) are almost always normal. While JME is accepted as one of the generalized epilepsy syndromes, some clinical and electroencephalographic (EEG) characteristics support local hyperexcitability of the cortex. In recent years, it has been emphasized that JME patients have trouble with the frontal lobe and visuospatial functions. Besides, they also have social adaptation and character issues more than the patients who have primary GTC seizures [2].

Various findings related to frontal lobe involvement in JME have also been detected in different neuroimaging studies conducted on the pathophysiology of the disease. In two different studies performed with MRI spectroscopy, it was stated that bilateral frontal and prefrontal N-acetyl-aspartate (NAA) levels were low in patients with JME, and the decrease in NAA in frontal areas paralleled the deterioration in frontal lobe findings in neuropsychological tests [3,4].

Therefore, our study was aimed to investigate the executive functions and personality traits in JME patients with neuropsychologic and neurocognitive scales to determine if there is frontal lobe dysfunction.

## 2. Materials and methods

### 2.1. Study population

The present study included 23 JME patients and 20 healthy controls. JME patients 18–40 years of age, who did not have a mental or systemic disorder, and were followed up in the outpatient neurology department were included. The patients who had GTC seizures in the last week and absence or myoclonic seizures on the last day were excluded from the study. When the patients were grouped according to the seizure types, patients with myoclonus and GTC seizures were in the majority (n: 12, 52.2%). The second most common was myoclonus, generalized tonic-clonic, and absence seizures (n: 9, 39.1%). They were divided into 3 groups; those who did not have a seizure in the last 1 year (Group 1 with 8 patients), those who had seizures only in the presence of a trigger factor in the last 1 year (Group 2 with 10 patients), and those who continued seizures without a trigger factor in the last 1 year (Group 3 with 5 patients). The patients who received monotherapy were using valproic acid (n: 15, 65.2%), lamotrigine (n: 4, 17.4%), and levetiracetam (n: 1, 4.3%) (87% in total), while the patients using polytherapy were under valproic acid and levetiracetam treatment (n: 3, 13%).

### 2.2. Clinical scales

Hospital anxiety and depression scale (HADS)

The HADS scale is designed to scan mood disorders in populations who suffer from medical disease. It is filled in by the patient, consists of 14 items and two subscales evaluate the levels of depression and anxiety [5].

Digit span test

A digit span test which is a subtest of Wechsler Adult Intelligence Scale-R (WAIS-R) and Wechsler Memory Scale-R (WMS-R) can be used to assess simple attention. This test has two subdivisions which are forward and backward digit spans. Forward span captures attention efficiency and capacity. Backward span is an executive task particularly dependent on working memory [6,7].

Stroop Color and Word Test (SCWT)

The SCWT test evaluates attention, cognitive control, and the ability of code-switching reaction and interference. The four different cards in this test have warnings, and the test measures the reaction of the subject [8].

The Temperament and Character Inventory (TCI)

The TCI is a 240-item self-administered questionnaire constructed to assess four temperaments (novelty seeking, harm avoidance, reward dependence, and persistence) and three character dimensions (self-directedness, cooperativeness, and self-transcendence) and contains 25 lower-order scores [9].

### 2.3. Statistical analyses

All statistical data were analyzed using the Statistical Program for Social Sciences 20.0 (SPSS). While the chi-square test was used for conducting the bivariate analysis of the differences between the patient groups. The *T*-test was performed in independent groups to compare the normally distributed quantitative data. Mann Whitney-U test was used for nonnormally distributed data. The Pearson correlation was used for comparing the relationship between two continuous variables. The Kruskal-Wallis test is a rank-based nonparametric test that can be used to determine if there are statistically significant differences between two or more groups of an independent variable on a continuous or ordinal dependent variable. Bonferroni correction was used as post hoc after Kruskal-Wallis test. Moreover, p ≤ 0.05 was considered statistically significant.

### 2.4. Ethics committee approval

The study protocol was approved by the Clinical Research Ethics Committee of Marmara University with the 09.2016.201 decision number on March 4, 2016. Additionally, all patients signed informed consent which was prepared according to the principles of the Declaration of Helsinki before the study.

## 3. Results

We enrolled 23 JME patients (17 females, 6 males) and 20 healthy control subjects (14 females, 6 males) in this study.

As shown in Table 1, no significant difference was observed between the patients and the control group with respect to gender, age, and education year (p: 0.775, 0.692, 0.292). Age at onset of epilepsy was 9–19 years (mean 14.78) and epilepsy duration was 2–36 years (mean 10.78).

Also, as shown in Table 2, no significant difference was seen between the depression and anxiety scores of the patients and the control group (p: 0.201, 0.647). Hence, there was no need to neutralize depression or anxiety impact in other neuropsychological tests.

Additionally, the measures of the mean forward and backward digit span were statistically significant between the patient and the control groups as shown in Table 3 (p: 0.016, 0.006 < 0.05). When we compared digit-span test scores according to the frequency of seizures with Kruskal-Wallis test with Bonferroni correction as shown in Table 4, we found that the 3rd group achieved significantly lower forward scores (p: 0.031< 0.05). Further, as presented in Table 5, the completion time of S1–5 parts and the total test were statistically significant and longer in the patient group than the control group (total time p: 0.0002 < 0.05). There was no significant relationship between epilepsy duration, digit-span, and Stroop test scores when analyzed with the Pearson correlation test (p < 0.05). Also as shown in Table 6, there was no significant relationship between Stroop test scores and seizure frequency when analyzed with the Kruskal-Wallis test with Bonferroni correction.

In addition, as shown in Table 7, cooperativeness-character subdimension (C1) scores were significantly lower in the patient group (p: 0.027 < 0.05).

## 4. Discussion

The intelligence levels and neurological examinations are usually normal in JME patients. However, as Janz and Christian who described the illness in 1957, pointed out, JME patients have a special characteristic profile like impulsive behavior, affective lability, can be easily influenced, lack of discipline, irresponsibleness, impatience, lack of self-confidence, juvenility, being indifferent to illness, etc. Anxiety and depression also occur frequently. Because of that, the necessity of determining that characteristic profile with neuropsychological scales has appeared and it is conceived that impulsive personality structure might be related to frontal lobe dysfunction. Of note, the therapeutical doses of most antiepileptic drugs do not affect attention and behavior except phenobarbital, gabapentin, and topiramate [2,10]. Since our patients were not using any of these drugs by chance, neutralizing the drug effect was not needed. Also, the drug levels of our patients using valproic acid were not at toxic levels, but we could not check the levels of other drugs. This situation might be another limitation of our study. Also, we know that background activities are normal in the EEG examinations of our patients, a more detailed study can be planned about whether there is a focal or generalized epileptiform activity and the relationship between the frequency of these activities and other neuropsychological test parameters.

The frontal lobe is especially associated with attention, planning, executive functions, psychomotor speed, and recalling. In our study, when digit-span test data were analyzed, the forward (attention and short-term memory) and backward (working memory and executive functions) digit scores were seen to be lower. While the Stroop test data were analyzed which measured selective attention, interference, and resistance to interference, the JME patients took longer to complete the test than the control group subject. Studies investigating cognition in JME showed average general intelligence; however, this parallels the disruption of verbal productivity, working memory, and a wide range of executive functions, with medium to large effect sizes and also influenced by semantic knowledge, reasoning, processing speed, and dexterity. However, the overall evidence for learning and memory deficits is conflicting [11]. Eventually, the results of our study correspond to the research that shows that the JME patients’ frontal lobe functions are affected.

Moschetta et al. used temperament end character inventory and found higher novelty-seeking and harm avoidance scores, lower self-directedness scores in 42 JME patients than the control group. In the same study, higher novelty-seeking scores significantly correlated with earlier age at the onset of epilepsy and higher frequency of myoclonic seizure [12]. In another study, JME patients with personality disorders were reported to have more difficulty in seizure control and worse functional performance when compared to those without these behavioral traits [13]. In our study, we believe that we could not find similar results because there were fewer JME patients who had well-controlled seizures with respect to the classification of Prasad et al. mentioned earlier [14]. On the other hand, we found the patient group had lower social acceptance scores (C1-cooperativeness lower order) which is known to be related to all categories of personality disorder [15]. Although low C1 scores have not been observed alone in JME patients in other studies, low cooperativeness scores in epilepsy patients are associated with perceived social support and low quality of life as mentioned in the study of Demirci et al. [16]. Further studies with a larger sample and detailed psychosocial assessment will be more explanatory about character issues in JME patients.

Subjects with JME, high levels of novelty-seeking, and impulsive behavior might, therefore, bear a genetic predisposition to dopamine pathway alteration. Ciumas and colleagues stated that reduced dopamine transporter binding in the frontal lobe and striatum along with impaired psychomotor speed, motor function, and attention could be related to the behavioral and cognitive problems in JME patients [17].

In conclusion, cognitive function and personality traits might be affected in JME patients. It is important to know whether our patients have these problems in order to manage the treatment process better and be aware of the potential stigma. Therefore, more studies involving neuroimaging, genetic, and neuropsychological evaluation are greatly awaited.

## Figures and Tables

**Table 1:** Demographic features and education year of the study groups.

	Patient (n: 23)	Control (n: 20)	p-value
Sex	6 males (26.1%)	6 males (26.1%)	0.775^a^
	17 females (73.9%)	14 females (70%)	
Age	25.27 ± 4.65	26.20 ± 5.79	0.692^b^
Education year	13 [5–15]	11 [8–15]	0.573^c^

a Chi-square test,

b Student t-test,

c Mann Whitney-U

**Table 2:** Comparison of anxiety and depression scale data of the study groups.

	Patient (n: 23)	Control (n: 20)	p-value
Anxiety	8.04 ± 3.65	6.60 ± 3.62	0.201^a^
Depression	4 [0–10]	5 [0–12]	0.241^b^

a Student t-test,

b Mann Whitney-U

**Table 3:** Comparison of digit-span test data of the study groups.

	Patient (n: 23)	Control (n: 20)	p-value
Forward	5.91 ± 1.73	7.25 ± 1.74	0.016^a^
Backward	4.87 ± 1.91	6.55 ± 1.85	0.006^a^

a Student t-test

**Table 4:** Analysis of digit-span test scores according to the frequency of seizures.

Seizure frequency	Group 1		Group 2		Group 3		p
	n = 8		n = 10		n = 5		
	Mean ± Std	Median [min–max]	Mean ± Std	Median [min–max]	Mean ± Std	Median [min–max]	
Forward	5.88 ± 1.36	5.50 [4–8]	6.70 ± 1.95	6 [4–10]^*^	4.40 ± 0.55	4 [4–5]^*^	0.031^a^
Backward	5.00 ± 1.93	4.50 [2–8]	5.50 ± 1.84	5.00 [3–8]	3.40 ± 1.52	3 [2–5]	0.181^a^

a Kruskal-Wallis test (with Bonferroni correction).

**Table 5:** Comparison of Stroop test data of the study groups.

TIME (s)	Patient (n: 23)	Control (n: 20)	p-value
S1	9.91 ± 2.02	7.65 ± 1.09	0.00004^a^
S2	10.57 ± 2.33	8.40 ± 1.31	0.001^a^
S3	13.09 ± 2.61	10.65 ± 1.89	0.001^a^
S4	16.22 ± 2.70	13.75 ± 2.86	0.006^a^
S5	24.78 ± 5.38	19.95 ± 4.97	0.004^a^
Total	74.56 ± 12.54	60.40 ± 10.00	0.0002^a^

a Student t-test

**Table 6:** Analysis of Stroop test scores according to the frequency of seizures.

Seizure frequency S-1	Group 1		Group 2		Group 3		p
	n = 8		n = 10		n = 5		
	Mean ± Std	Median [min–max]	Mean ± Std	Median [min–max]	Mean ± Std	Median [min–max]	
	9.88 ± 2.36	9 [7–15]	9.50 ± 2.07	9 [7–13]	10.80 ± 1.30	10 [10–13]	0.209^a^
S-2	10.75 ± 3.15	10.50 [7–17]	10.10 ± 1.97	9 [8–13]	11.20 ± 1.64	11 [10–14]	0.534^a^
S-3	12.13 ± 2.23	12.5 [9–15]	13.50 ± 2.95	13 [9–18]	13.80 ± 2.49	13 [12–18]	0.545^a^
S-4	15.63 ± 2.26	15.5 [11–18]	15.90 ± 2.73	15.5 [12–20]	17.80 ± 3.19	17 [14–22]	0.485^a^
S-5	26.00 ± 5.66	26.5 [17–33]	22.40 ± 5.06	21.5 [17–34]	27.60 ± 4.34	28 [22–34]	0.108^a^

a Kruskal-Wallis test (with Bonferroni correction).

**Table 7:** Comparison of TCI scale data of the study groups.

	Patient (n: 23)	Control (n: 20)	p-value
NS1	6.39 ± 1.64	6.15 ± 2.43	0.702^a^
NS2	3 [1–6]	4 [2–10]	0.097^b^
NS3	4.65 ± 1.97	5.15 ± 2.08	0.426^a^
NS4	4.57 ± 1.67	4.85 ± 1.72	0.586^a^
HA1	5 [2–11]	6 [0–9]	0.314^b^
HA2	3.91 ± 1.50	3.65 ± 2.13	0.640^a^
HA3	3.43 ± 2.19	2.75 ± 2.10	0.303^a^
HA4	3.83 ± 1.87	3.25 ± 1.97	0.332^a^
RD1	7.65 ± 1.67	7.00 ± 1.81	0.226^a^
RD2	4.78 ± 1.81	4.55 ± 1.73	0.670^a^
RD3	2 [0–4]	3 [1–5]	0.670^b^
P	5.26 ± 2.03	4.75 ± 2.34	0.447^a^
SD1	4.74 ± 1.71	4.65 ± 2.34	0.887^a^
SD2	6 [2–8]	5 [3–7]	0.514^b^
SD3	3 [1–5]	3.5 [0–5]	0.164^b^
SD4	5.57 ± 2.15	6.05 ± 2.35	0.484^a^
SD5	9 [6–11]	9 [6–12]	0.675^b^
C1	7 [4–8]	8 [4–8]	0.027^b^
C2	4 [2–7]	5 [3–7]	0.156^b^
C3	4 [3–6]	5 [3–7]	0.083^b^
C4	8 [4–10]	9 [1–10]	0.681^b^
C5	6 [2–8]	6 [2–8]	0.450^b^
ST1	7.00 ± 2.45	5.95 ± 2.76	0.194^a^
ST2	4.91 ± 2.02	5.70 ± 1.69	0.177^a^
ST3	7.57 ± 3.16	5.90 ± 2.85	0.078^a^

a Student t*-*test,

b Mann Whitney-U

(Temperament: NS-Novelty seeking, HA-Harm avoidance, RD-Reward dependence, P-Persistence. Character: SD-Self-directedness, C-Cooperativeness, ST-Self-transcendence).
